# Correction: Searching across-cohort relatives in 54,092 GWAS samples via encrypted genotype regression

**DOI:** 10.1371/journal.pgen.1011149

**Published:** 2024-02-21

**Authors:** The PLOS Genetics Staff

## Notice of Republication

Incorrect versions of S1 Text, S3 Fig, S4 Fig, S5 Fig, S6 Fig, S6 Table and S7 Table were published in error. This article was republished on January 18, 2024 to correct for this error. Please download this article again to view the correct version.
