# Supplementary figures and images for: Searching across-cohort relatives in 54,092 GWAS samples via encrypted genotype regression
Source: PLoS Genet. 2024 Jan 11;20(1):e1011037. doi: 10.1371/journal.pgen.1011037 (PMC10783776; doi:10.1371/journal.pgen.1011037)

**A****I**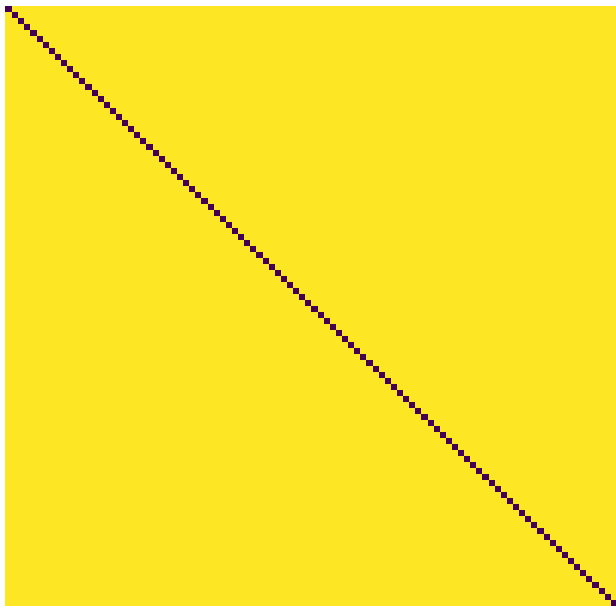**B** **$SS^T$** 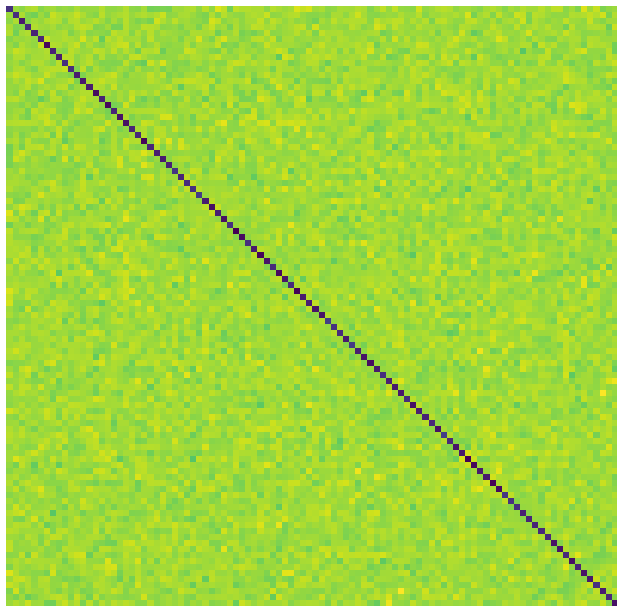**C** **$X_1X_2$** 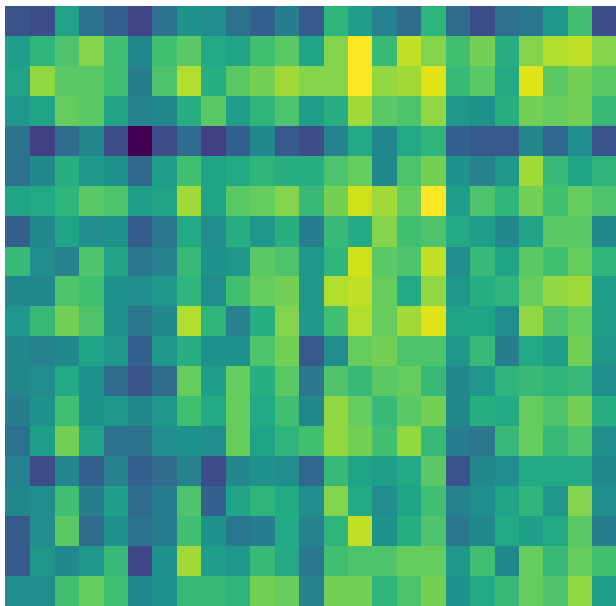**D** **$X_1SS^TX_2$** 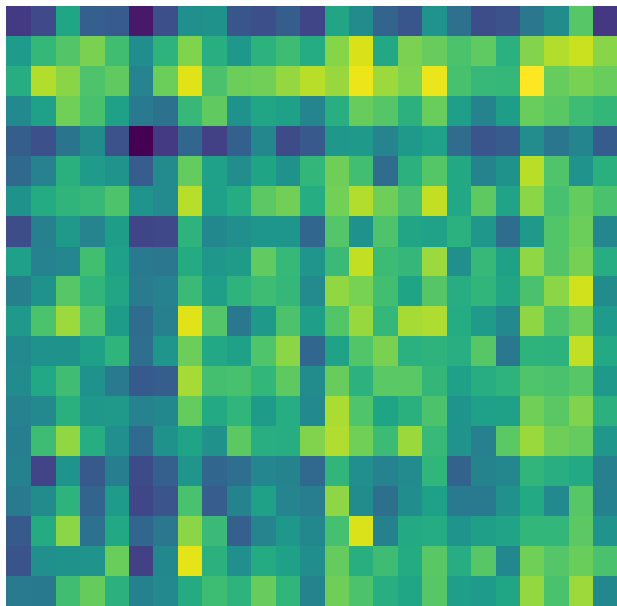

Supplement: S1 Fig — We generate a random matrix Sm×k sampling from N(0,1/k) and plot SST (B) against an identity matrix (A). We also generate two small populations containing 20 and 25 individuals, respectively. Their genotype matrices are noted as X1 and X2, and plot the matrix multiplication product X1X2T before (C) and after encryption X1SSTX2T (D). The column number for the random matrix is k = 500 and the number of SNPs is m = 100. (PDF) [file pgen.1011037.s002.pdf]

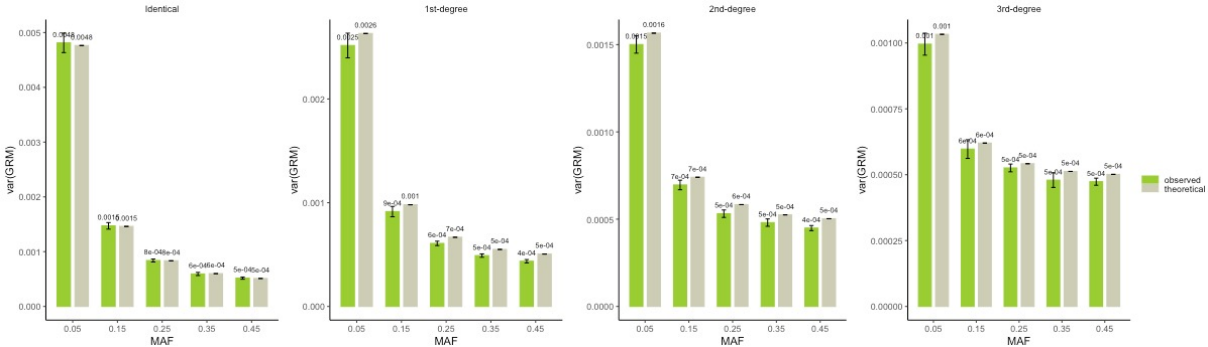

Supplement: S2 Fig — To testify the variance of GRM under the assumption of binomial distribution, we simulated 1,000 pairs of different degrees of relatives, and 2,000 markers with same MAF from 0.05 to 0.45 per increase in 0.1. We compared the observed variance of relatedness with the theoretical relatedness in 10 repeats. (PDF) [file pgen.1011037.s003.pdf]

1201528 SNPs

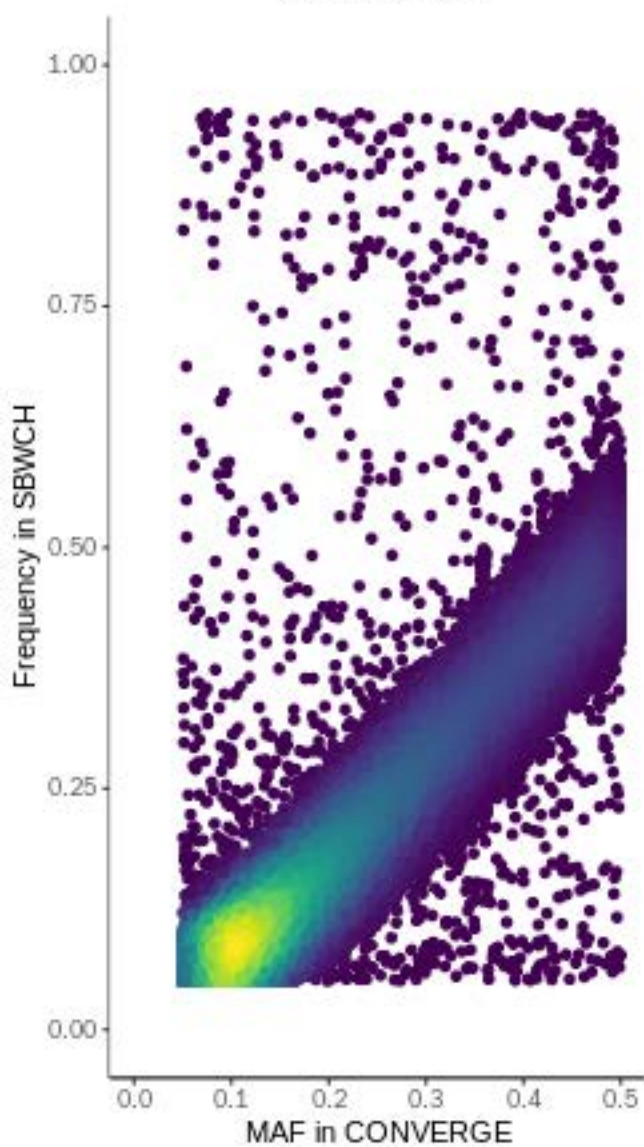

256944 SNPs

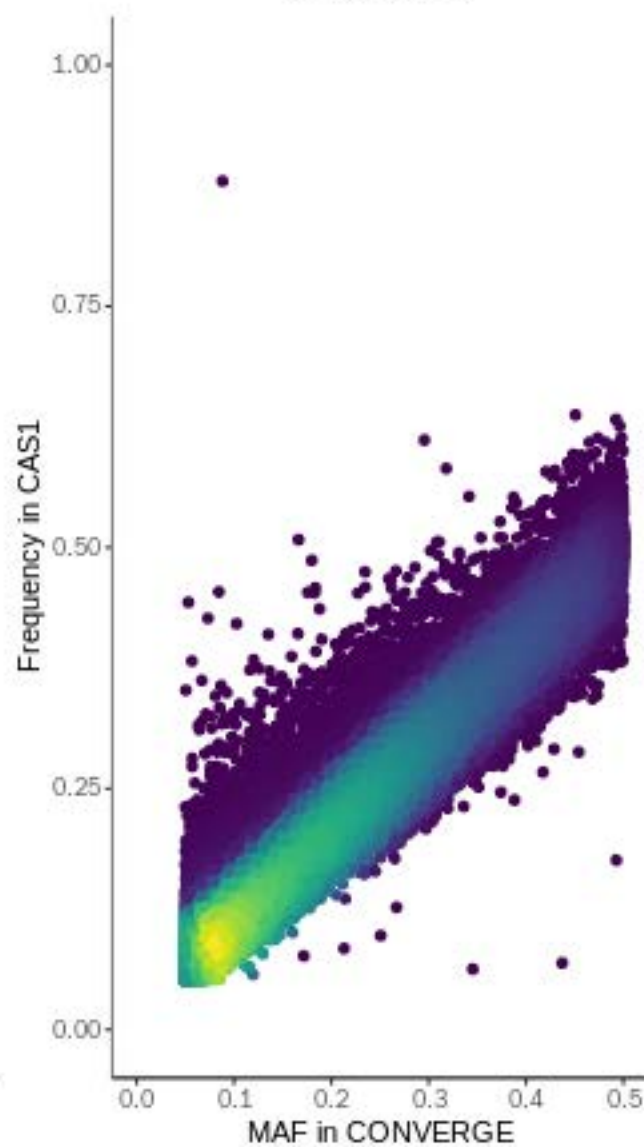

256932 SNPs

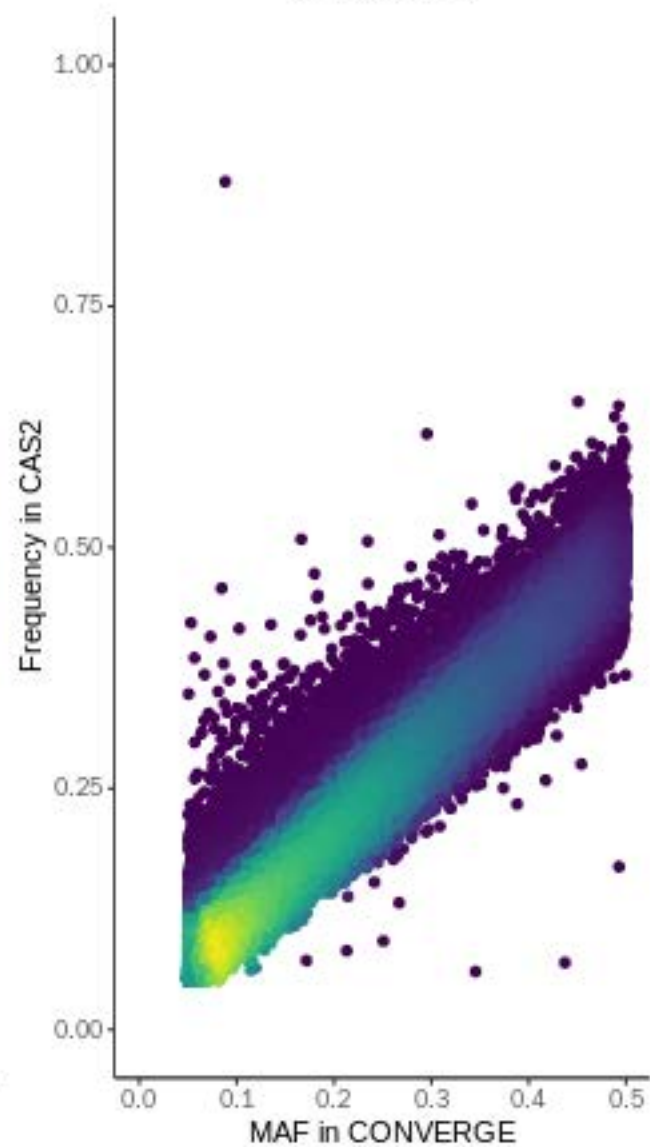

310596 SNPs

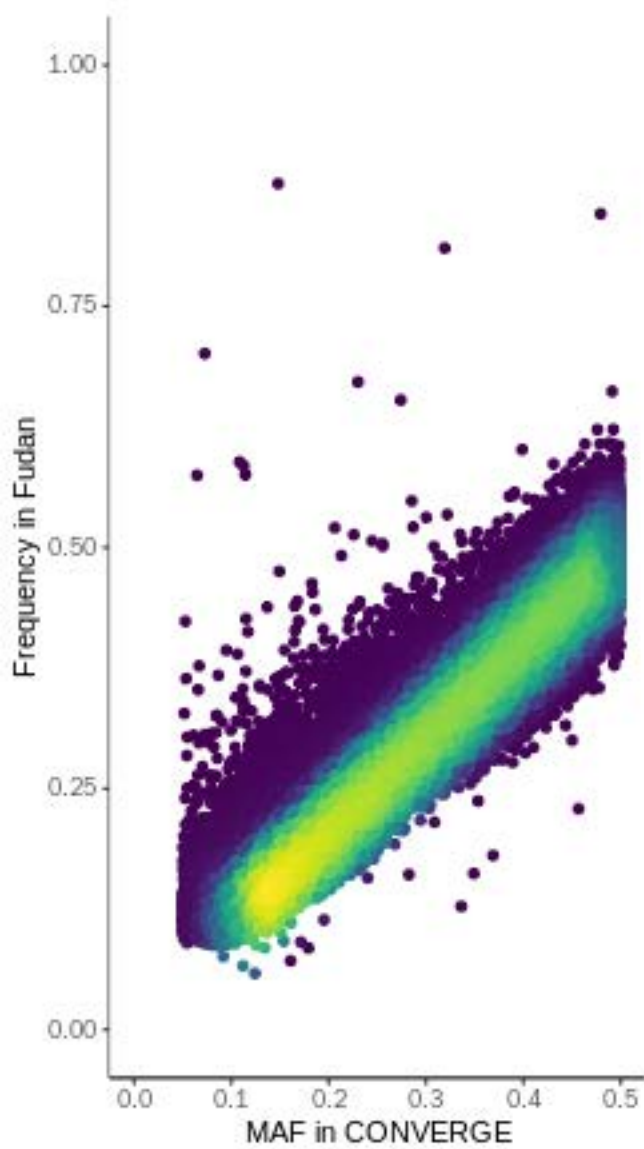

285897 SNPs

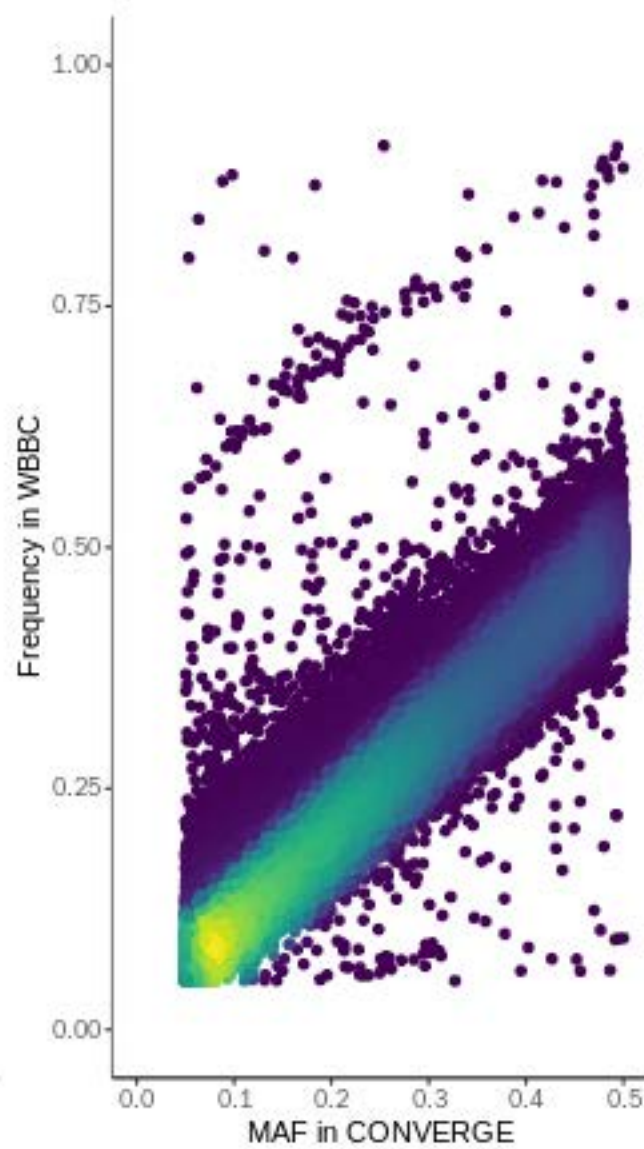

Supplement: S3 Fig — Comparison of MAF in CONVERGE with the frequency of the same allele in each cohort. Each hexagonal bin is colored according to the number of markers falling in that bin. (PDF) [file pgen.1011037.s004.pdf]

Shared SNP quality

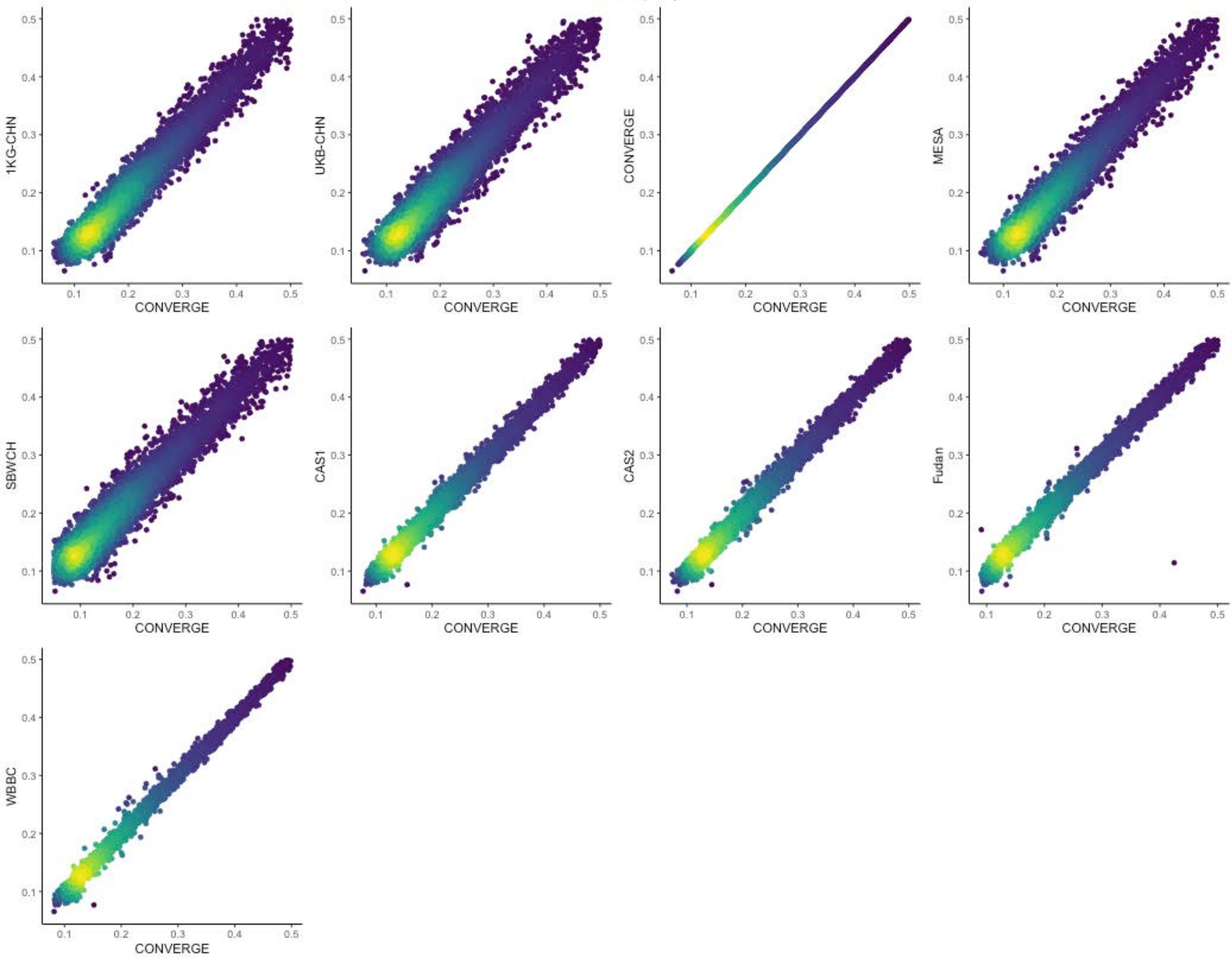

Supplement: S4 Fig — Comparison of MAF in CONVERGE with the frequency of the same allele in each Chinese cohort, considering 7,009 overlapping SNPs only. Each hexagonal bin is colored according to the number of markers falling in that bin. (PDF) [file pgen.1011037.s005.pdf]

**1KG-CHN**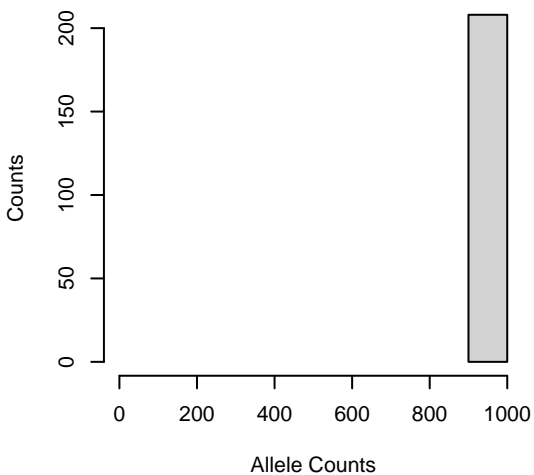**UKB-CHN**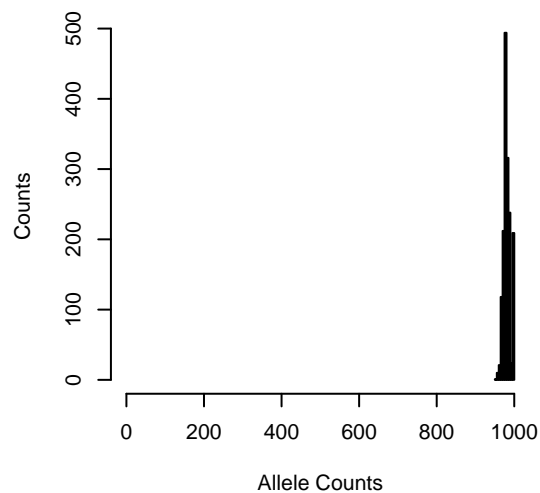**CONVERGE**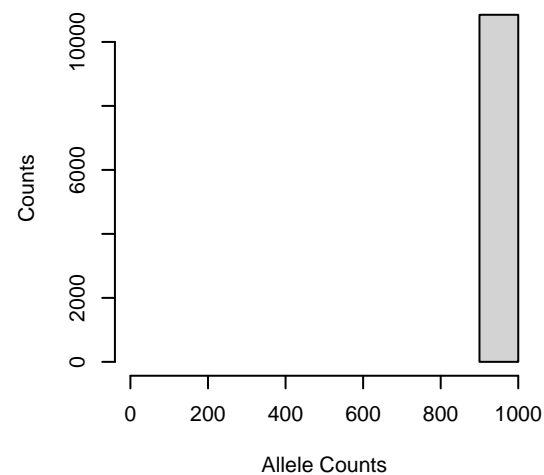**MESA**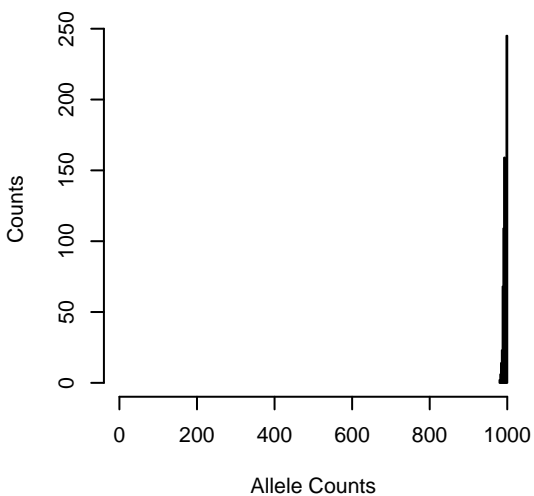**SBWCH**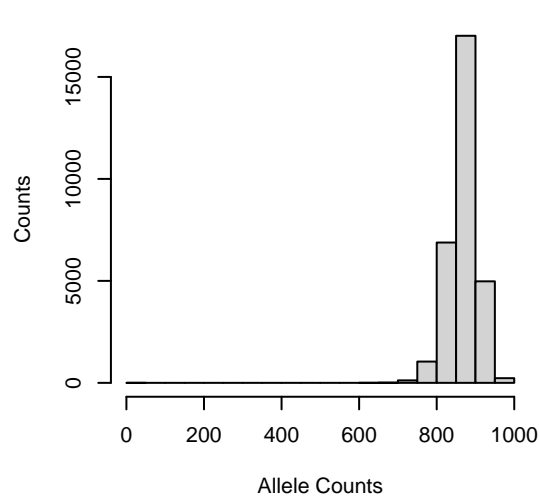**CAS1**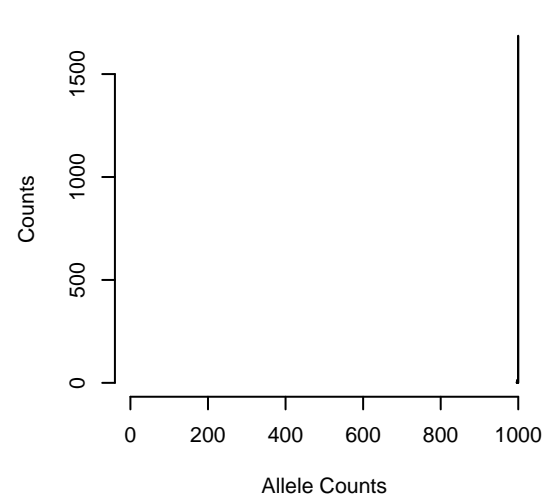**CAS2**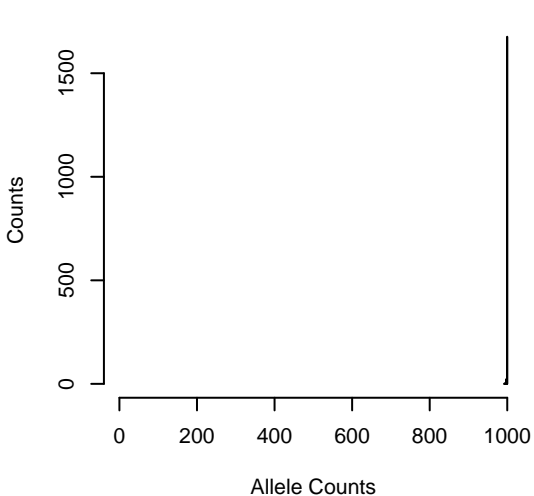**Fudan**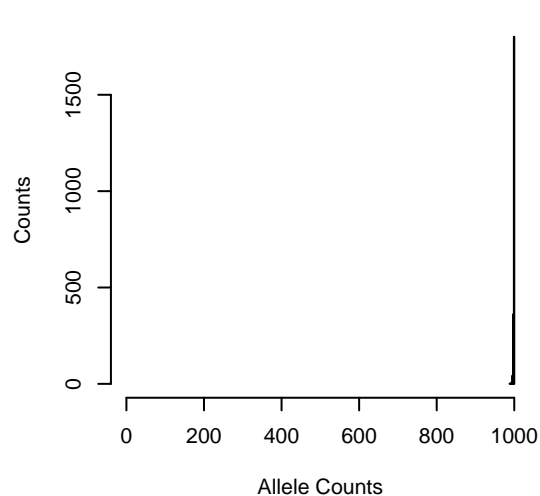**WBBC**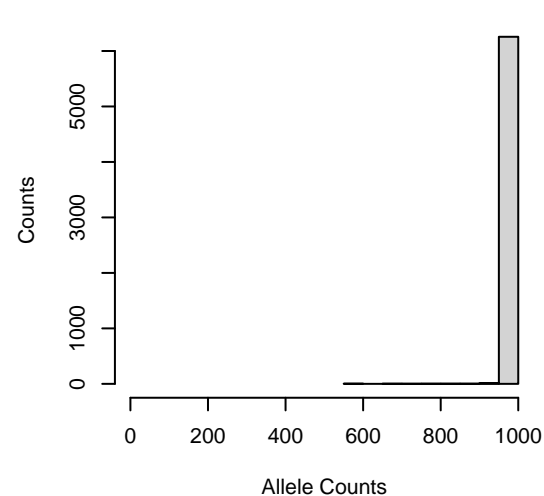

Supplement: S5 Fig — Distributions of non-missing allele counts in each cohort. Maximum allele counts = 2m = 1,000. (PDF) [file pgen.1011037.s006.pdf]

density

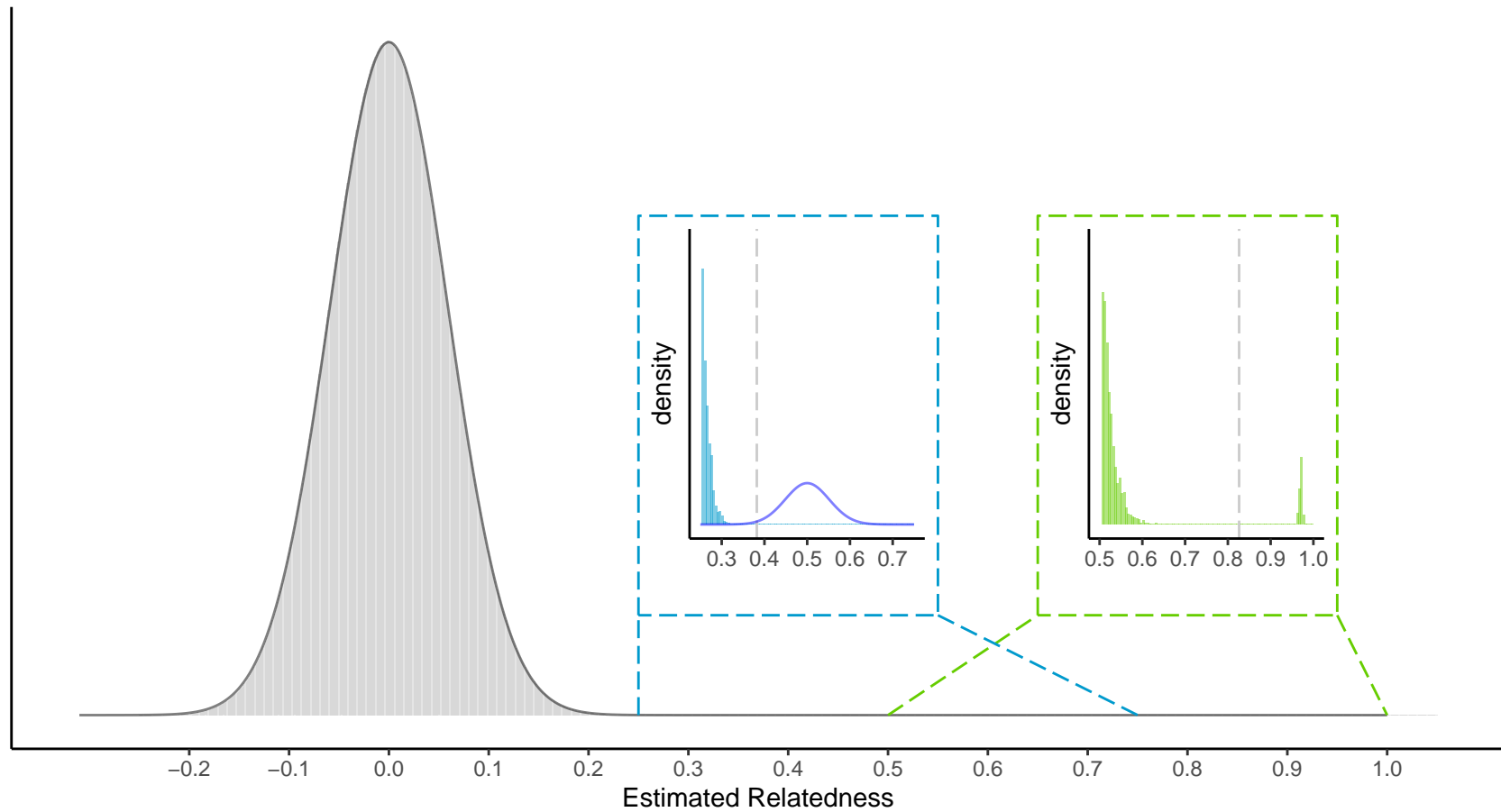

Supplement: S6 Fig — The histogram shows all estimated relatedness using encG-reg between SBWCH and WBBC, most of which are unrelated pairs and the theoretical probability density function is given as the normal distribution N(0,1me+1k1) (grey solid curve). The inset histogram on the left shows the estimated relatedness around 0.5 and the theoretical probability density function is given as the normal distribution N(θr,1−θr2me+1−θr2k1) (blue solid curve). The threshold (grey dot line) for rejecting H0 was calculated by z1−α/N1me+1k1. The inset histogram on the right shows the estimated relatedness around 1. The threshold (grey dot line) for rejecting H0 was calculated by z1−α/N1me+1k0. Here we included 208 controls merged from 1KG-CHN. me = 477, k0 = 70, k1 = 710, N=930,140,004. (PDF) [file pgen.1011037.s007.pdf]
